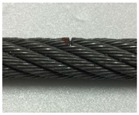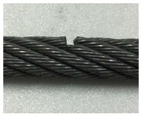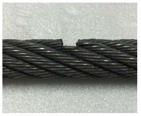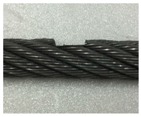# Magnetic Flux Leakage Sensing and Artificial Neural Network Pattern Recognition-Based Automated Damage Detection and Quantification for Wire Rope Non-Destructive Evaluation

**DOI:** 10.3390/s18010109

**Published:** 2018-01-02

**Authors:** Ju-Won Kim, Seunghee Park

**Affiliations:** School of Civil, Architectural Engineering and Landscape Architecture, Sungkyunkwan University, Suwon 16419, Korea; malsi@nate.com

**Keywords:** magnetic flux leakage, steel wire rope inspection, signal processing, damage quantification, artificial neural network

## Abstract

In this study, a magnetic flux leakage (MFL) method, known to be a suitable non-destructive evaluation (NDE) method for continuum ferromagnetic structures, was used to detect local damage when inspecting steel wire ropes. To demonstrate the proposed damage detection method through experiments, a multi-channel MFL sensor head was fabricated using a Hall sensor array and magnetic yokes to adapt to the wire rope. To prepare the damaged wire-rope specimens, several different amounts of artificial damages were inflicted on wire ropes. The MFL sensor head was used to scan the damaged specimens to measure the magnetic flux signals. After obtaining the signals, a series of signal processing steps, including the enveloping process based on the Hilbert transform (HT), was performed to better recognize the MFL signals by reducing the unexpected noise. The enveloped signals were then analyzed for objective damage detection by comparing them with a threshold that was established based on the generalized extreme value (GEV) distribution. The detected MFL signals that exceed the threshold were analyzed quantitatively by extracting the magnetic features from the MFL signals. To improve the quantitative analysis, damage indexes based on the relationship between the enveloped MFL signal and the threshold value were also utilized, along with a general damage index for the MFL method. The detected MFL signals for each damage type were quantified by using the proposed damage indexes and the general damage indexes for the MFL method. Finally, an artificial neural network (ANN) based multi-stage pattern recognition method using extracted multi-scale damage indexes was implemented to automatically estimate the severity of the damage. To analyze the reliability of the MFL-based automated wire rope NDE method, the accuracy and reliability were evaluated by comparing the repeatedly estimated damage size and the actual damage size.

## 1. Introduction

Recently, elevators for convenience of movement and transportation have become essential facilities inside of buildings, particularly with the development of high-rise buildings, and the installation of elevators has been rapidly increasing all over the world. In addition, cranes and lifts are essential pieces of equipment for transporting materials on construction sites.

In various industrial facilities, steel wire rope, which has a high strength and high flexibility, is a key mechanical element used for power transmission and is widely used because it has the advantages of reliability and efficiency.

Wire ropes fully support the load of structures or cargo, so damage to a wire rope can lead to great risks. However, local cross-sectional damage in a wire rope can occur due to aging caused by long-term use, corrosion caused by the external environment, damage due to unexpected mechanical movement, and local defects due to friction with peripheral devices, etc. These small defects can expand quickly because of the tension in the wire rope, which can lead to the lifting structure falling apart or other structural failure. However, such damage is not easily detected due to certain properties of the wire rope, such as its complicated cross section and long length; and thus some wire ropes are being used in very dangerous conditions in situ [1,2].

Additional, due to the features mentioned above, the remaining service life of wire ropes cannot be accurately predicted, so the ropes are regularly replaced. In particular, it is estimated that more than 70% of ropes have been replaced despite the fact that there is no problem with the strength, and there is a recurring economic loss as a result. Thus, it is very important to detect initial defects in wire ropes at the early stages to prevent both accidents and avoid economic losses.

Currently, wire rope inspection relies almost entirely on manual inspection methods along with visual inspection. This requires a lot of time and money, and there is a high probability of human error because it is diagnosed is based on an individual’s decision. Visual inspection also has a fatal disadvantage in that it cannot inspect local flaws or internal corrosion.

To overcome these drawbacks, non-destructive testing has recently been introduced to check the state of wires, but only up to a limit. Techniques such as radiographic testing and ultrasonic testing (referred to as representative non-destructive testing methods) have the potential to reduce the risk of radiation exposure, but they are associated with low inspection efficiency and problems with wave propagation due to the geometry of the complicated wire ropes [2].

Therefore, magnetic testing can be a very useful method for detecting defects in wire ropes because such wire ropes are ferromagnetic materials that are easily magnetized, and most non-destructive testing methods for wire rope inspections primarily use magnetic sensors. In this method, a magnetic field is applied to a wire, and a magnetic sensor detects any changes in the magnetic flux or leakages of the magnetic field from a defective portion of the wire [3,4,5].

Magnetic sensors have the advantages of excellent reliability and reproducibility, and excellent diagnostic performance can be expected when combined with signal processing technology [6,7,8,9].

Magnetic sensors vary in type and are used in accordance with the characteristics of the target structure considering any possible damage [10]. Wire ropes are ferromagnetic and continuous, and have a complicated cross section in which several wires are twisted. In addition, the type of damage that occurs in the wire rope is characterized by disconnection, corrosion, and collapse of the shape, most of which occurs locally. Further, corrosion and abrasion damage, which can occur globally, the degree of degeneration of the corrosion and abrasion damage is progressed to leads to disconnection of the wire.

Considering these characteristics, the magnetic flux leakage (MFL) method was selected as a suitable test method for wire rope in this study. This is because the MFL method has been effectively applied to diagnose local damage in continuous ferromagnetic structures such as pipes and rails, and it has the advantage of high-speed non-contact diagnosis [11,12,13].

Currently, most wire rope diagnostic techniques using the MFL technique employ a one-dimensional raw magnetic flux signal. However, there is a restriction in that the damage must be judged via subjective signal analysis by a professional, and thus such methods are not widely used.

To overcome these limitations, this study aimed to develop an automatic damage assessment method based on MFL that quantitatively assesses the degree of damage by objectively determining whether or not the damage has occurred automatically without expert intervention.

To accomplish this, magnetic flux data measured from 8-channel MFL sensor heads were processed by filtering and the Hilbert transform method, outlier analysis was performed by comparing the results with a threshold using the GEV distribution, and the existence of damage was objectively determined.

Assuming damage was identified, damage indexes were created to quantify the MFL signals by extracting the magnetic characteristics of the MFL signals. Finally, an artificial neural network (ANN)-based pattern recognition technique using extracted damage indexes was developed, and the size of the damage in the wire rope was automatically and objectively estimated.

## 2. Theoretical Background

### 2.1. Magnetic Flux Leakage-Based Damage Detection Technique

**Figure 1:**
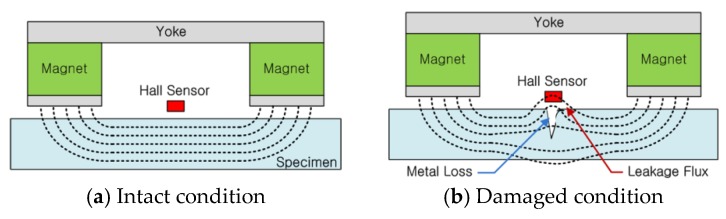
Schematic of the MFL method [9].

Any magnetized ferromagnetic material can be considered as a magnet. The magnetic field spreads out when it encounters a small air gap created by the defect, as the air cannot support as much magnetic field per unit volume when compared to the magnet. When the field spreads out, it starts to leak out of the material, and this is called magnetic flux leakage. Before measuring the magnetic flux leakage, specimens must reach saturated magnetization conditions by applying a field large enough to cause the magnetic flux to effectively leak out. To establish the magnetic flux in the material to be inspected, a strong permanent magnet is used to magnetize the specimen in this study. When no damage is present, the magnetic flux in the specimen remains uniform, as illustrated in Figure 1a. In contrast, flux leakage occurs when damage due to local defects has occurred, as shown in Figure 1b.

**Figure 2:**
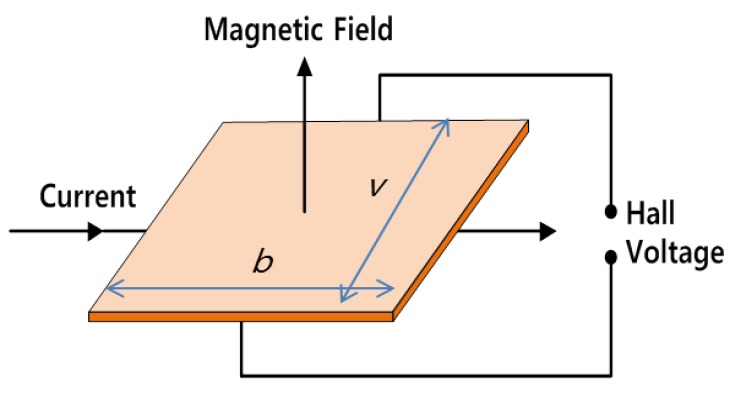
Schematic of the Hall effect [10].

Magnetic flux leaks out of the metal specimen near the damaged areas. Sensors that can detect this flux leakage are placed between the poles of the magnet. The sensors then generate a voltage signal proportional to the magnetic flux leakage [10,14]. In this study, Hall sensors that operate based on the Hall effect were used to capture the MFL signal, as illustrated in Figure 2.

When a magnetic field (*B*) is applied to a plate, an electron moving through the magnetic field experiences a force, known as the Lorentz force, which is perpendicular to both to the direction of motion and the direction of the field. The response to this force then creates a Hall voltage [10]. This Hall voltage can be measured using a data acquisition (DAQ) system and can then be used to examine the condition of the target structure.

### 2.2. Signal Processing for Improving Signal Quality

**Figure 3:**
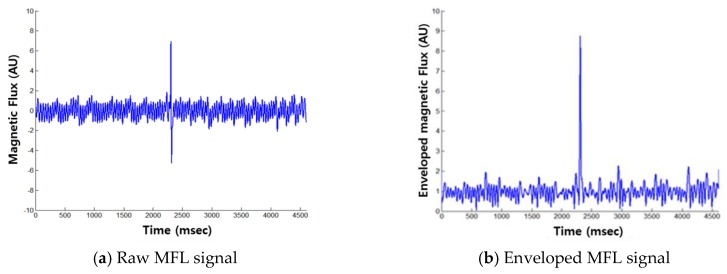
Effect of the enveloping process [9].

Signal processing techniques, such as low-pass filtering and offset correction, were performed to improve signal resolution after measuring the magnetic flux. After the de-noising process was performed, the enveloping process was carried out to determine flux leakage to improve the accuracy of damage detection using the characteristics of the magnetic flux signal [9]. Samples of the raw MFL signal and the enveloped MFL signal are shown in Figure 3.

This enveloping process based on the Hilbert transform was performed to bring out the MFL signal due to damage [15]. The envelope using the Hilbert transform can be obtained based on the instantaneous amplitude, and it is useful for analyzing abnormal signals generated by defects in a time series signal. This enveloping process can help reveal important information about the signal by reducing meaningless information, which is used to improve damage detection. In addition, it is helpful for comparing the damage with a threshold value for decision making during damage detection, and additional damage indexes can be extracted from envelope signals to quantify the damage level.

### 2.3. Establishing Threshold Levels Using the GEV Distribution for Damage Detection

After obtaining the magnetic flux signal, the appropriate threshold that distinguishes between the normal and damaged conditions needs to be established. In this study, a 99.99% confidence level threshold of the normal condition was established using the generalized extreme value (GEV) distribution. The GEV distribution is the limit distribution of the properly normalized maxima of a sequence of independent and identically distributed random variables according to the extreme value theorem [16]. The GEV distribution was therefore used as an approximation to model the maxima of long sequences of random variables. The generalized extreme value distribution has a cumulative distribution function, that is shown in Equation (1):

(1) $$F(x;\mu ,\sigma ,\xi )=\exp\{-{\left[1+\xi (\frac{x-\mu }{\sigma })\right]}^{-1/\xi }$$

for 1 + *ξ*(*x* − *μ*)/*σ* > 0, where *μ* ∈ R is the location parameter, *σ* > 0 is the scale parameter, and *ξ* ∈ R is the shape parameter [16]. When the magnetic flux signal exceeds the calculated threshold value, the signal is determined to be within a damaged range.

### 2.4. Damage Quantification Using MFL Signal Based Damage Indexes

Typically, two kinds of damage indexes that can be extracted from raw MFL signals have been used to quantify the MFL signal to estimate the amount of damage [17,18].

**Figure 4:**
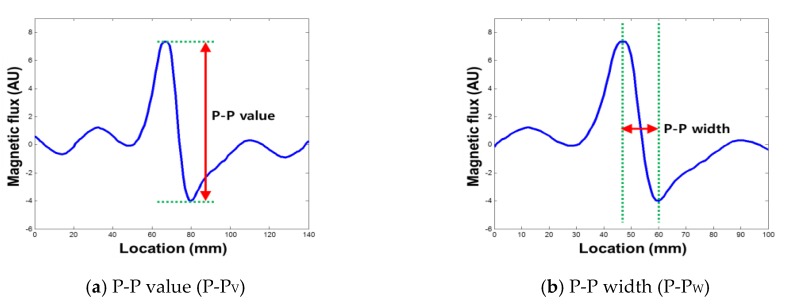
Common damage index for an MFL signal [9].

The peak to peak value (P-P value: P-P_V_) shown in Figure 4a is used to represent the y-component (amplitude) of an MFL signal and is known to represent the depth of damage. On the other hand, the x-component (width) of the MFL signal is represented by the peak to peak width (P-P width: P-P_W_), as shown in Figure 4b. In this study, four types of new damage indexes were extracted from the relationship between the enveloped MFL signal and the threshold, which are utilized to quantify the damage level [9].

**Figure 5:**
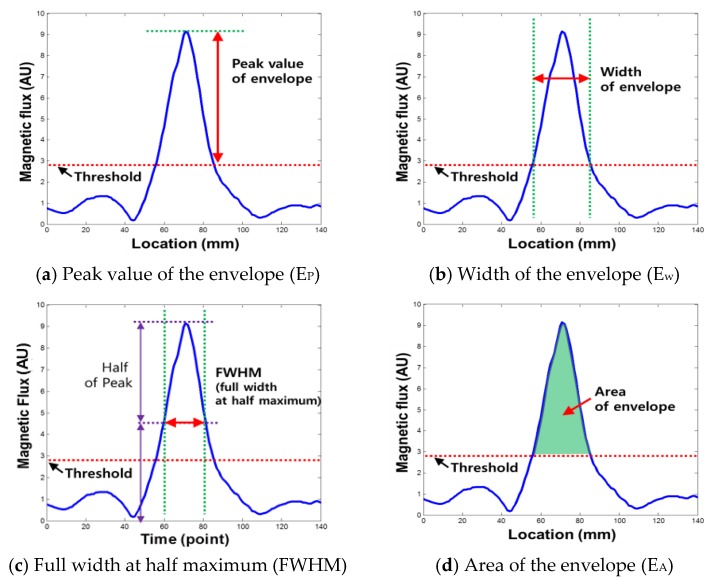
New damage indexes using the relationship between the envelope signal and the threshold [9].

First, the maximum peak of the enveloped signal that exceeds the threshold was extracted and was named the ‘peak value of envelope (E_P_)’ as shown in Figure 5a. This measurement more effectively represents the level of damage when the MFL signal is saturated; this is because the saturated part of an MFL signal can be restored using the enveloping process since the trends in the remaining signal are reflected in the enveloped signal.

Also, the width of the envelope (E_W_) was extracted to supplement the P-P width by calculating the range where the envelope exceeds the threshold, which was then used to represent the x-component of the enveloped MFL signal, as shown in Figure 5b. The E_W_ extracted from under the peak is generally larger than the P-P width, in accordance with the triangular shape [9].

However, when the peak is too large or too small, E_W_ cannot reflect the magnetic properties of the peak due to the fixed, relatively low threshold. To deal with this limitation, the FWHM (full width at half maximum) was applied to stably represent the width of the peak. To extract the FWHM, the width value at half height of the peak was indexed when the peak was obtained from a signal, as shown in Figure 5c [19].

In addition, the area of the envelope (E_A_) was extracted by integrating the amplitude of the signal in the excess range considering, the shape of the envelope signal, as shown in Figure 5d. Even if the height and width are the same, the shape of the peak may change. Thus, the area of the envelope can effectively represent the total energy of the magnetic flux leakage, as it can reflect the shape of the enveloped MFL signal. 

These extracted damage indexes can be independently used to quantify the damage. However, since they have mutually complementary relationships, the accuracy of damage evaluation can be improved by using different combination of these indexes, such as developing complex multi-dimensional damage indexes or utilizing various parameters for pattern recognition.

### 2.5. ANN Based Pattern Recognition for Damage Quantification

An artificial neural network (ANN) can be used to make an approximation function by learning from collected data, and it can objectively classify unknown data based on this approximation function [20]. The ANN consists of a number of processing elements that are connected to form layers of neurons, although the networks may be complex. The missing links between sets of inputs and outputs were found by determining the optimal synaptic weights, based on the available training data of the inputs and outputs [21]. In this research, various damage indexes were extracted from the MFL signals and were used as training data to train the ANN classifier for the purposes of estimating the damage in a wire rope.

A supervised multi-layer feed-forward ANN with backpropagation is typically employed. The Levenberg-Marquardt (LM) algorithm, which is similar to the Newton method [22], is used for back propagation in ANN learning. The root mean squared error (RMSE) was used as the performance index in this study. In this research, various damage indexes were extracted from the MFL signal and were used as training data to teach the ANN how to estimate the damage in a wire rope.

## 3. Experimental Study

### 3.1. Experimental Setup & Procedure

**Figure 6:**
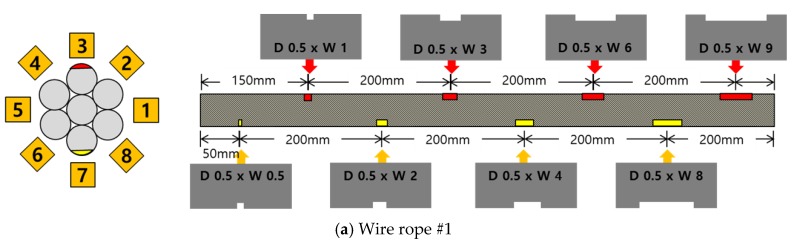

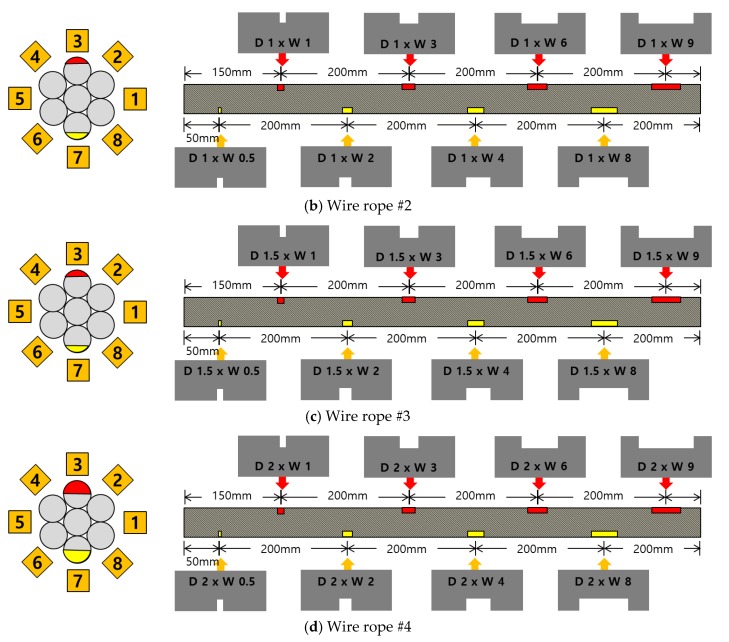
Specification of the wire rope specimens.

A series of experimental studies was carried out to examine the capabilities of the proposed damage detection and quantification technique. To perform the experiments, five steel wire-rope specimens with a 10 mm diameter and 800 mm length were prepared. An intact wire rope was prepared and magnetic flux signals were measured from this wire rope, which were then used to establish the threshold for the outlier analysis. Four kinds of artificial damaged steel wire specimens were prepared for quantitative analysis according to the size of the damage, as shown in Figure 6 and Table 1.

As shown in Table 1 and Table 2 and Figure 6, stepwise formations of various damage sizes were considered for all wire ropes (#1 to #4), for a total of 32 different types of local damage. In each wire rope, eight types of damage with a width ranging from 0.5 mm to 9 mm were machined, with four in the upper parts of the rope and four in the lower parts with a 10 cm spacing. The depths of the damage were 0.5 mm for wire rope #1, 1 mm for wire rope #2, 1.5 mm for wire rope #3, and 2 mm for wire rope #4.

**Figure 7:**
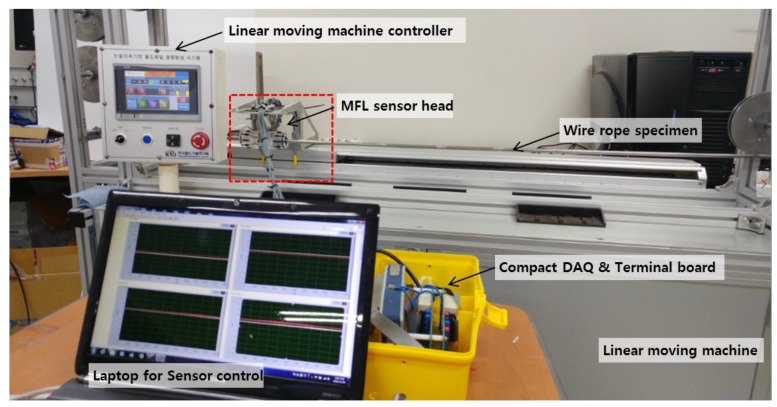
Experimental setup.

In other words, the change in MFL signal was determined according to the change in damage width. The changes in MFL signal were also analyzed for various damage depths, which was accomplished by comparing the wire ropes (#1–#4) with different depths. The test setup for measuring MFL signals was composed of an MFL sensor head, a compact DAQ, and a terminal board, as shown in Figure 7.

The MFL sensor head moves linearly along the fixed wire rope specimen with a constant velocity of 2 m/s to measure MFL signals using a linear moving machine. The data acquisition equipment, which consists of a terminal board and a compact DAQ, measures 8 magnetic flux signals simultaneously at the MFL sensor head.

**Figure 8:**
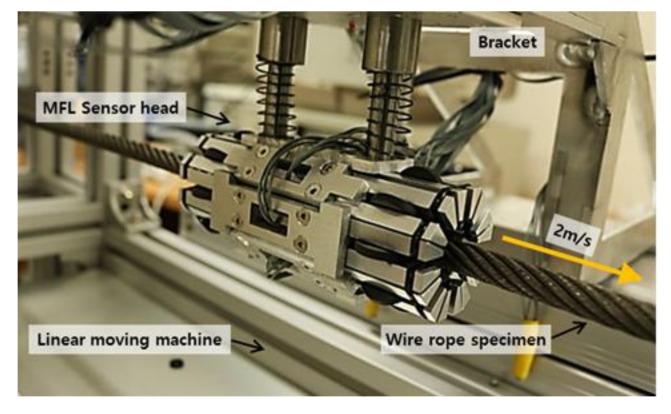
8-channel MFL sensor head.

The MFL sensor head is composed of 8 channels of the sensor module, and each sensing module contains a Hall sensor and a permanent magnet yoke that can independently measure the magnetic flux signal. Eight sensing modules were circumferentially arranged in a circular configuration to measure signals from the entire cross section of the wire rope, as shown in Figure 8.

Signals were repeatedly measured 40 times at each specimen, and the sampling rate was 10 kHz. The measured signals were then processed to facilitate effective damage detection throughout signal processing and the enveloping process based on the Hilbert transform (HT).

### 3.2. MFL Based Damage Detection Results

**Figure 9:**
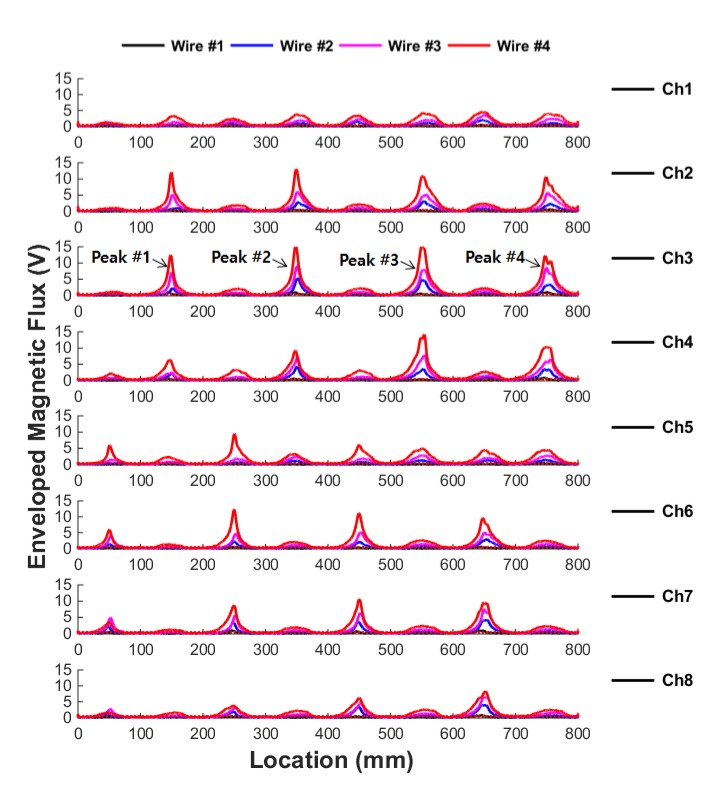
Overlapped graphs of the enveloped MFL signals for wire ropes #1–#4.

After signal processing, the enveloped MFL signals measured from wire ropes #1–#4 are displayed by overlapping in Figure 9.

Figure 9 shows that leakage signals exceeding the threshold were generated at 150, 350, 550 and 750 mm, where the actual damage is located. It was also confirmed that leakage signals were generated at 50, 250, 450 and 650 mm, where damage was located. This shows that the detection of damage in the wire rope is possible through detection of the MFL signal.

The most sensitive sensing channel for upper damages is channel 3, which is nearest to the upper damage, and channel 7 is most sensitive for lower damages. A relatively weaker MFL signal was detected in the neighboring sensing channels. This shows that the closer the damage is to the sensing channel, the better the detection capability of the signal, indicating that the circumferential direction of the damage can be deduced using this method.

### 3.3. Quantitative MFL Signal Analysis Using Damage Indexes

#### 3.3.1. Analysis and Quantification of the Leakage Flux Signal with Increasing Damage Depth

MFL signals collected from wires #1–#4 with different depths of damage were compared to analyze the change in patterns of the MFL signals with increasing damage depth. In this research, a quantitative analysis was conducted using the MFL signals measured from the top four damaged regions by utilizing the most sensitively measured signals from channel 3.

**Figure 10:**
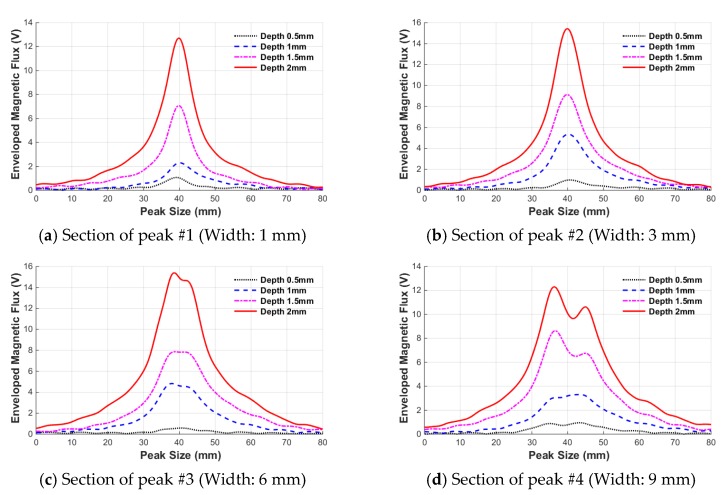
Variation of the enveloped MFL signal according to damage depth.

It can be seen from Figure 10 that the magnitude of the envelope signal increases with the depth of the defects in all peaks sections. In general, the height of the peak, which is known to be affected by the depth, increased stepwise with increasing depth, while the width of the envelope, which is known to be closely correlated with the damage width, increased with increasing depth. Comparing each peak section, however, it is seen that the size of the peak varies with the width, even though the depth damage is the same; therefore, it is confirmed that the height of the peak is not determined by the depth of the damage.

**Figure 11:**
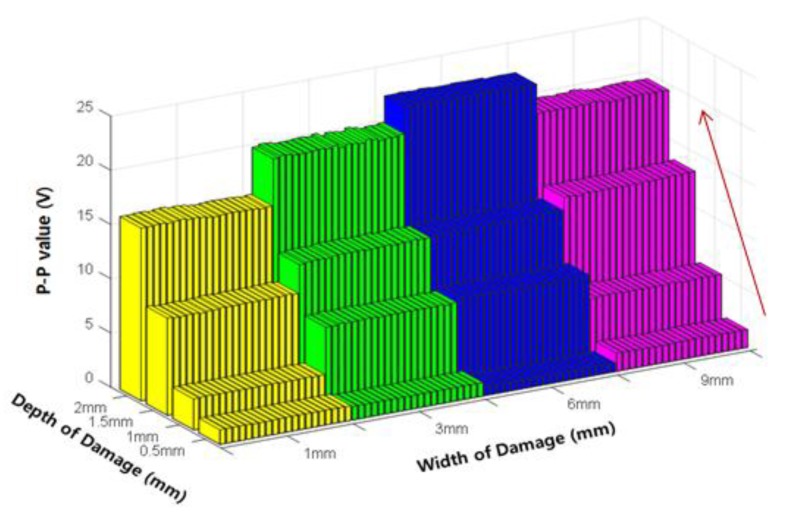
Variation of the P-P value (P-P_V_) according to the damage depth.

Next, the changes in envelope signal were quantitatively extracted according to the damage depth by using the damage indexes introduced in Section 2.4. First, the most representative index of the MFL signal, the P-P value (P-P_V_), is presented in Figure 11. As previously seen in the peak signal in Figure 10, the P-P value increased as the damage depth increased in accordance with the width of the damage.

**Figure 12:**
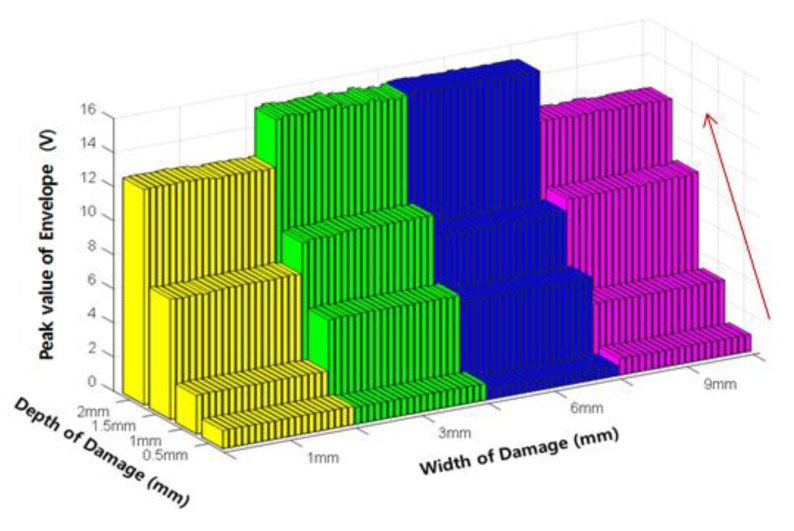
Variation of the peak value of envelope (E_P_) according to the damage depth.

Figure 12 shows the extraction results of the peak value of the envelope using the relationship between the envelope signal and the threshold value. The peak value of envelope (E_P_), which is similar to the P-P value, steadily increased with increasing damage depth.

**Figure 13:**
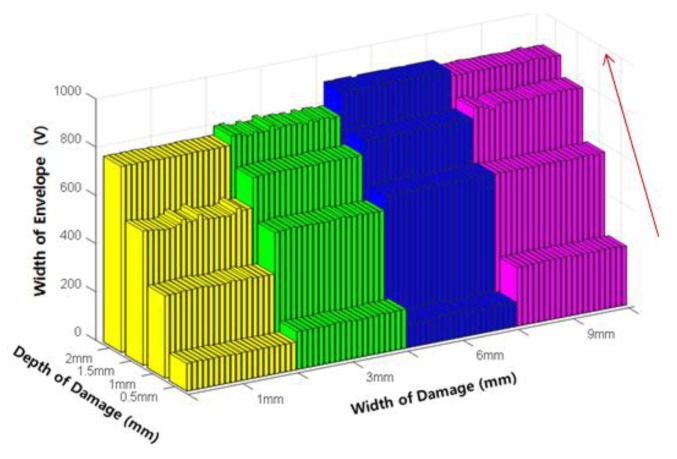
Variation of the width of envelope (E_W_) according to the damage depth.

Figure 13 below shows the results of extracting the width of envelope (E_W_), which is a damage index developed to estimate the original damage width. The width of the envelope exhibits a positive relationship with increasing damage depth, and was therefore determined to be appropriate both for estimating the damage width, and the damage depth.

**Figure 14:**
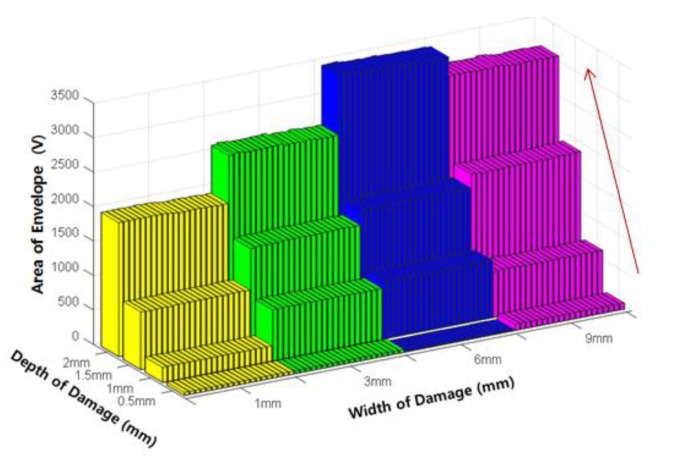
Variation of the area of envelope (E_A_) according to the damage depth.

The area of the envelope (E_A_) is shown in Figure 14. The peak value of envelope and the width of the envelope were determined based on how the envelope increased with increasing damage depth. Therefore, the area of the envelope also increased with increasing damage depth, exhibiting a large increase in magnitude close to the product of the two exponents.

An analysis of the changes in the damage indexes according to damage depth showed that all four indexes, i.e., the P-P value, peak value of the envelope, width of the envelope, and area of the envelope, increased with increasing damage depth. Therefore, these indexes were used with the ANN to estimate the damage depth.

#### 3.3.2. Analysis and Quantification of the Leakage Flux Signal with Increasing Damage Width

**Figure 15:**
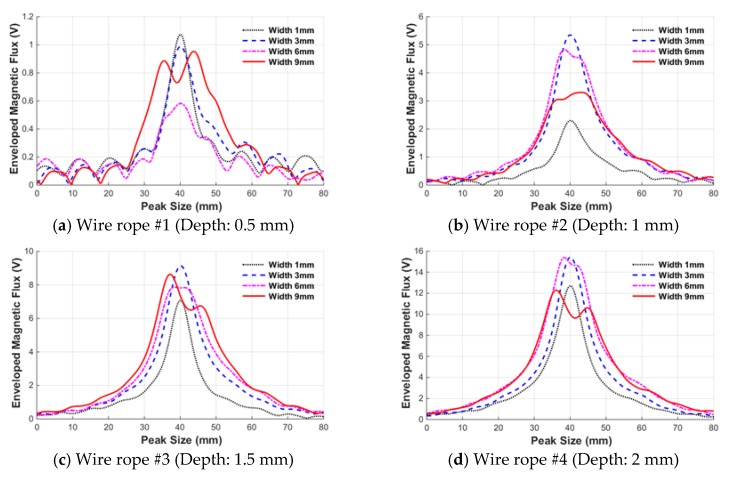
Variation of the enveloped MFL signals according to the damage width.

Figure 15 shows data for wire rope specimens with four different widths overlaid on top of each specimen. These data were used to examine the characteristics of the MFL signal according to damage width.

In the case of wire rope #1, the peak was not clear because the damage depth was very small (0.5 mm), and therefore no intuitive pattern for the height and width of the peak due to the damage width was identified. However, for a damage depth of 1 mm, the width of the peak increased gradually from 1 mm to 6 mm with increasing damage width. However, when the damage width was extended from 6 mm to 9 mm, the peak was distorted and the height of the peak decreased. Additionally, the width of the peak did not significantly increase either, and unlike damage depth, there was no consistent pattern, thus confirming that the characteristics vary slightly depending on the size of the peak.

The change in leakage flux signal with increases in the damage width was investigated by quantifying the damage index determined using various characteristics of the peak.

**Figure 16:**
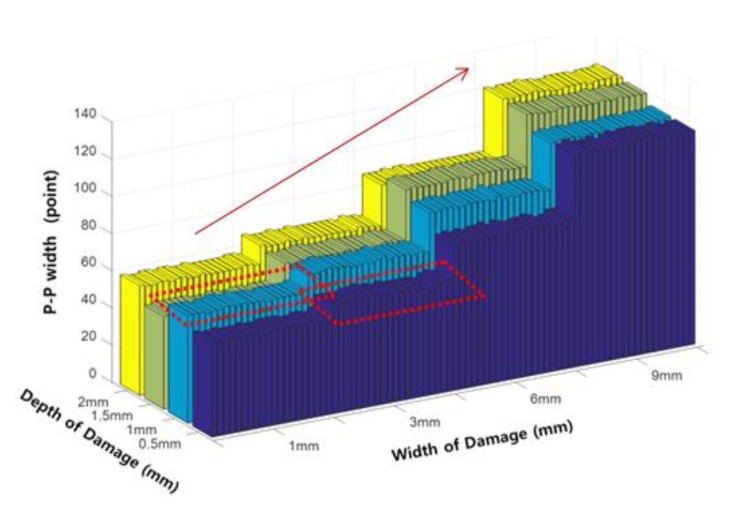
P-P width (P-P_W_) according to the damage width.

First, the P-P width (P-P_W_), commonly known as a representative index that reflects the width of the damage, was extracted and is shown in Figure 16.

**Figure 17:**
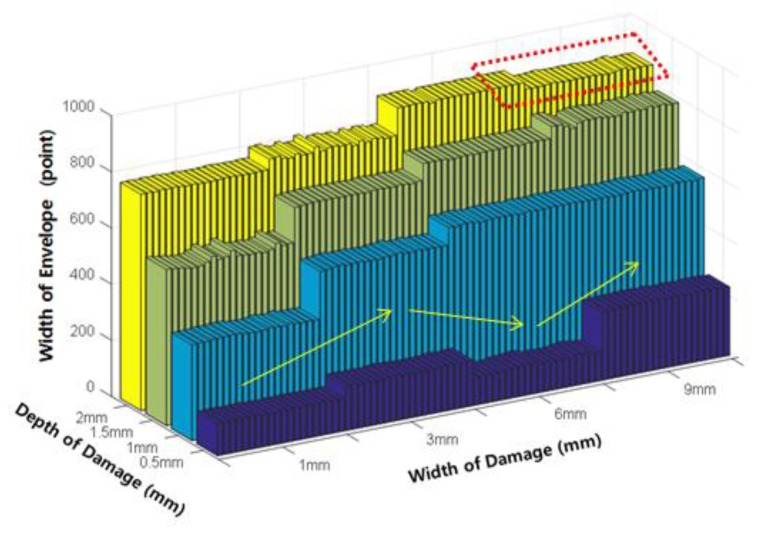
Width of the envelope (E_W_) according to damage width.

Figure 16 shows that the P-P width at each depth experienced a gradual increase with increasing damage width. However, complete separation does not occur because the P-P width has a value similar to the P-P widths at damage widths and depths of (i) 1 mm and 1 mm, and (ii) 3 mm and 0.5 mm, respectively. Figure 17 shows the width of the envelope (E_W_) as a function of the damage width variation.

The index decreased at a depth of 0.5 mm without increasing stepwise at a width of 6 mm. This is because the peak size itself is too small, as can be seen by comparing with the peaks in Figure 15. In addition, the index decreased in the section where the depth increased from 6 mm to 9 mm at a damage depth of 2 mm; this was concluded to be an error caused by peak distortion. In addition, the overall height of the peak decreased and the threshold value was smaller than the height of the peak.

**Figure 18:**
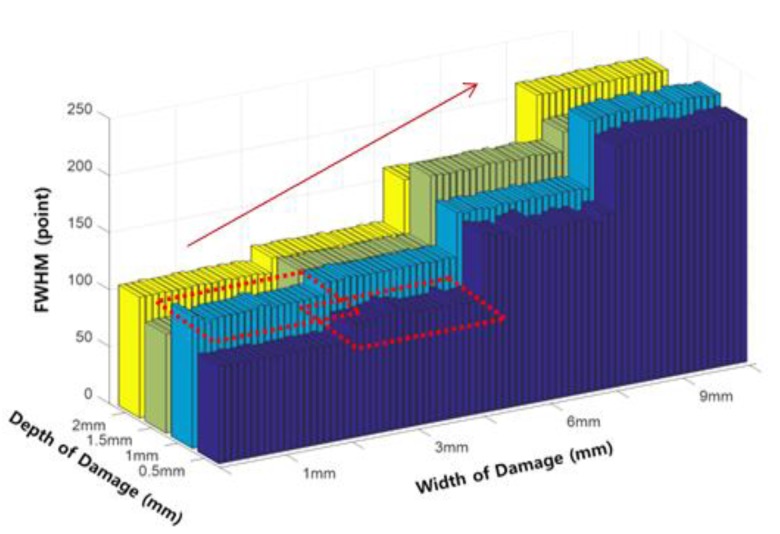
FWHM according to the damage width.

To compensate for the effects of size reduction, the FWHM extracted at the half height of the peak was used instead, as shown in Figure 18.

Extracted FWHM values exhibited a positive correlation with increasing width, similar to the P-P value. However, the values for the 1 mm width index and 3 mm width index were still not completely separated. However, since it was confirmed that changes in the index follow a certain pattern, this index can be effectively used when constructing the pattern recognition algorithm.

### 3.4. ANN Based Wire Rope Damage Size Estimation

#### 3.4.1. Procedure of ANN Based Damage Size Estimation

**Figure 19:**
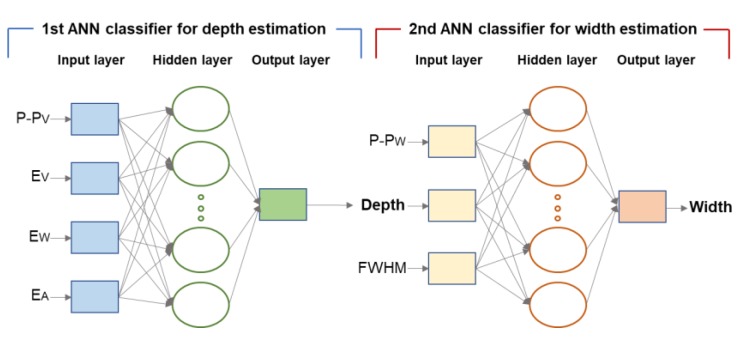
Two-step ANN pattern recognition process.

To estimate the depth and width of damage in the wire ropes, an ANN based pattern recognition technique was applied in this study. Estimation of the damage size was performed using a two-step ANN pattern recognition process as shown in Figure 19. After estimating the depth of the damage using the 1st ANN classifier, the width was estimated by re-reflecting the estimated depth value. Among the various ANN algorithms, the Levenberg–Marquardt algorithm was used to estimate the damage size in this study.

#### 3.4.2. Depth Estimation of Wire Rope Damage Using the ANN

Various damage indexes were used for training the ANN classifier for damage depth estimation. Based on the previous section, it was confirmed that the P-P value, peak value of the envelope, area of the envelope, and width of the envelope have positive relationships with increasing damage depth. Therefore, the ANN classifier was trained using all four kinds of damage indexes.

**Figure 20:**
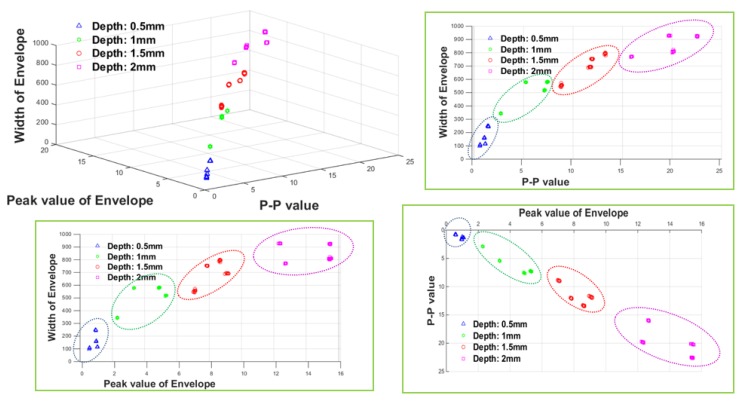
Three-dimensional distribution of the damage indexes for training the ANN for depth estimation.

The distribution of the learning data obtained by mapping it into three-dimensional space is displayed in Figure 20. In reality, four damage indexes were used, but only three indexes were used in the following graphs due to dimensional restrictions of visualization.

As shown in Figure 20, each of the damage indexes reflected the change in depth, and even when the damage indexes were mapped into three-dimensional space, they were clearly classified according to the damage depth.

As mentioned above, the ANN classifier was trained using four kinds of damage indexes, each extracted from 40 data points according to the damage depth.

**Figure 21:**
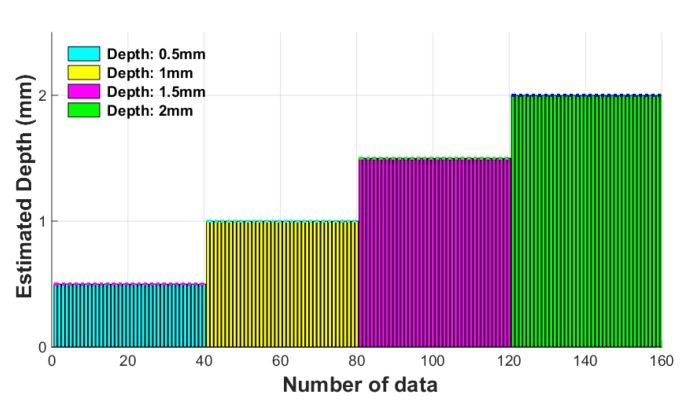
Estimated depth value using the ANN classifier.

To verify the performance of the ANN classifier, the damage indexes were extracted in the same way using the 40 test data collected under the same conditions, which were then substituted into the learned ANN classifier to estimate the depth of the damage. Figure 21 shows the results of the estimated damage depth according to the ANN classifier.

Estimation of the damage using the ANN is shown in Figure 21, which accurately estimates the depth of damage for each of the four types, each with a difference of 0.5 mm at every step. The error between the 40th estimated times of each step was also close to zero. Therefore, this ANN based depth estimation algorithm can estimate the damage depth with high accuracy for evaluating the condition of the wire rope.

#### 3.4.3. Width Estimation of Wire Rope Damage Using the ANN

Next, an algorithm for estimating the width of the damage via pattern recognition techniques using the ANN as well as damage depth estimation was investigated. The learning method for the ANN is similar to the method of damage depth estimation. Previously, the depth and width of the damage already confirmed that the damage indexes are sensitive to each other. Thus, the damage indexes used for depth estimation as well as other combinations of damage indexes were used for ANN learning for width estimation.

The FWHM and P-P width showed clear increasing patterns as the damage width increased. Therefore, this combination is a useful index for ANN classifier learning for the estimation of damage width.

**Figure 22:**
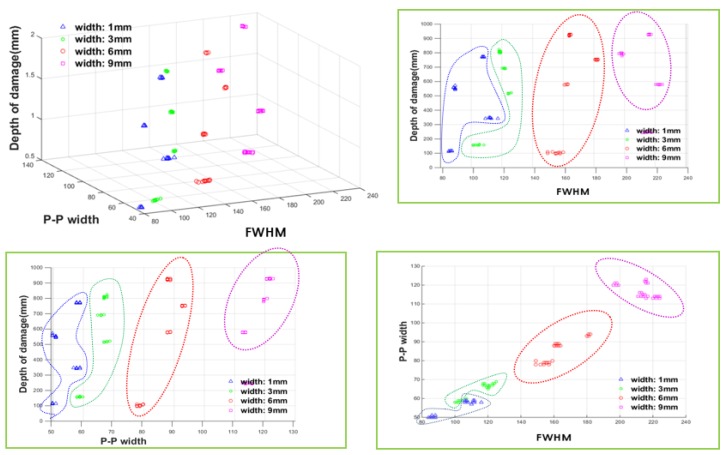
Three-dimensional distribution of the damage indexes for training the ANN for width estimation.

In addition, for the MFL signal, it was already confirmed that the depth of damage can also affect the damage index in terms of the damage width. Therefore, the ‘estimated depth value’, which was previously constructed during the damage depth estimation step using the ANN, was used as an auxiliary index for considering the damage depth. Therefore, P-P width, FWHM, and estimated depth value were used as the ANN learning data for width estimation. These are presented in three-dimensional space in Figure 22.

Figure 22 shows the distribution of the damage indexes for training the ANN for width estimation. There is an ambiguous boundary between the widths of 1 mm and 3 mm, but it is generally clustered according to increasing damage width.

As shown above, 40 sets of learning data were used for each damage width, and the ANN classifier was trained for width estimation.

**Figure 23:**
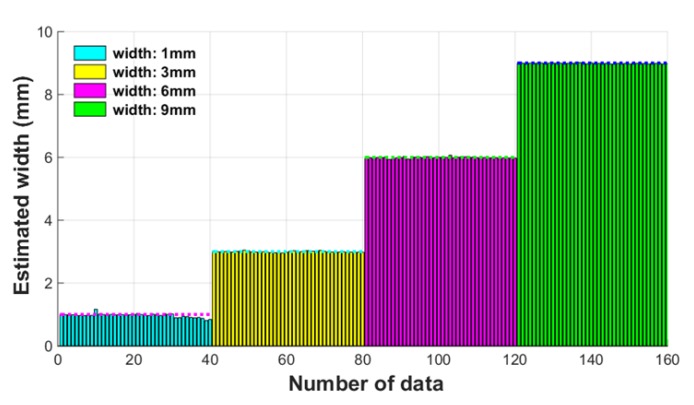
Estimated width value using the ANN classifier.

Subsequently, in order to verify the performance of the ANN classifier with regard to width estimation, 40 data per damage width were collected under the same experimental conditions as the training data used to the classifier. The results of the damage width estimation by the ANN classifier are shown in Figure 23.

All 40 of the estimates for damage width closely matched the damage width of the actual wire rope. There was an error of less than 0.1 between the measurement intervals, as can be confirmed by Figure 23, and such a small estimation error is negligible. Therefore, we confirmed that highly accurate damage width estimation was possible through an ANN based pattern recognition algorithm.

Therefore, it is expected that effective quantitative wire rope inspection can be provided through application of the MFL based NDE technique and the ANN pattern recognition technique presented in this research.

## 5. Conclusions

The MFL-based NDT technique was used to detect damage in steel wire ropes. An MFL sensor head was fabricated and a series of experimental studies were performed to verify the feasibility of the proposed technique. In addition, damage indexes were extracted to quantify the size of the damage. An ANN-based pattern recognition method using the extracted damage indexes was used to automatically estimate the amount of damage. This approach to wire rope NDE was confirmed through the following observations:

(1) Magnetic flux leakage was detected at locations with actual damage by using a Hall sensor located near the damage.
(2) The MFL signals at the damaged areas became more apparent via the enveloping process based on the Hilbert transform.
(3) Envelopes of the MFL signal exceeded the thresholds based on the GEV distribution around areas with actual damage.
(4) Damage indexes were extracted to quantify the MFL signals; these damage indexes can classify the damage size according to increases in damage size.
(5) Four types of damage indexes based on the relationship between the envelope signal and the threshold were proposed. These damage indexes can improve the accuracy of quantification of the damage size.
(6) Two-step ANN based pattern recognition was applied to estimate the depth and width of the damage. The ANN classifier was trained using multi-dimensional damage indexes extracted from the MFL signals; the trained ANN classifier can successfully estimate the size of damage with little error.

Overall, these results demonstrated that the proposed damage detection and quantification method using MFL sensors and an ANN classifier is capable of diagnosing defects in steel wire ropes. This approach will be complemented and validated by further research performed on various types of damage and environments.

In addition, it is expected that the proposed wire rope NDE method can be utilized as an advanced inspection tool for real-time in situ wire rope monitoring in combination with the Internet of Things and robot technologies.

## Figures and Tables

**Table 1:** Specification of the damage for wire ropes #1 and #2.

No.	Direction	Specification of the Damage							
Wire #1	Upper	Damage #1-2		Damage #1-4		Damage #1-6		Damage #1-8	
		Location	150 mm	Location	350 mm	Location	550 mm	Location	750 mm
		Depth	0.5 mm	Depth	0.5 mm	Depth	0.5 mm	Depth	0.5 mm
		Width	1 mm	Width	3 mm	Width	6 mm	Width	9 mm
		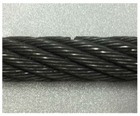		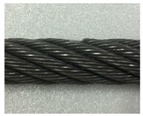		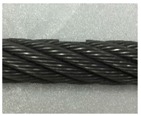		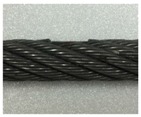	
	Under	Damage #1-1		Damage #1-3		Damage #1-5		Damage #1-7	
		Location	50 mm	Location	250 mm	Location	450 mm	Location	650 mm
		Depth	0.5 mm	Depth	0.5 mm	Depth	0.5 mm	Depth	0.5 mm
		Width	0.5 mm	Width	2 mm	Width	4 mm	Width	8 mm
		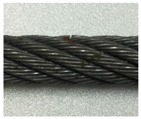		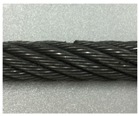		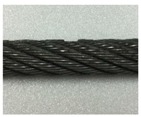		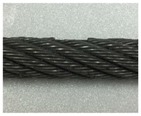	
Wire #2	Upper	Damage #2-2		Damage #2-4		Damage #2-6		Damage #2-8	
		Location	150 mm	Location	350 mm	Location	550 mm	Location	750 mm
		Depth	1 mm	Depth	1 mm	Depth	1 mm	Depth	1 mm
		Width	1 mm	Width	3 mm	Width	6 mm	Width	9 mm
		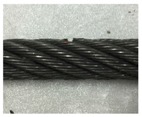		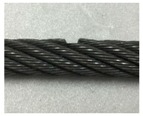		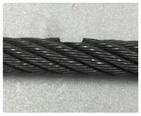		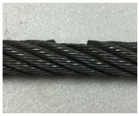	
	Under	Damage #2-1		Damage #2-3		Damage #2-5		Damage #2-7	
		Location	50 mm	Location	250 mm	Location	450 mm	Location	650 mm
		Depth	1 mm	Depth	1 mm	Depth	1 mm	Depth	1 mm
		Width	0.5 mm	Width	2 mm	Width	4 mm	Width	8 mm
		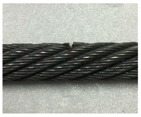		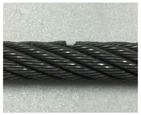		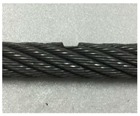		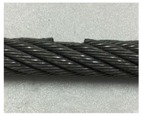	

**Table 2:** Specification of the damages for the wire rope #3 and #4.

No.	Direction	Specification of the Damage							
Wire #3	Upper	Damage #3-2		Damage #3-4		Damage #3-6		Damage #3-8	
		Location	150 mm	Location	350 mm	Location	550 mm	Location	750 mm
		Depth	1.5 mm	Depth	1.5 mm	Depth	1.5 mm	Depth	1.5 mm
		Width	1 mm	Width	3 mm	Width	6 mm	Width	9 mm
		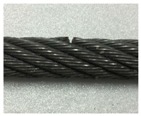		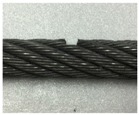		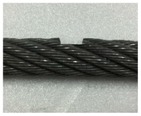		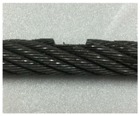	
	Under	Damage #3-1		Damage #3-3		Damage #3-5		Damage #3-7	
		Location	50 mm	Location	250 mm	Location	450 mm	Location	650 mm
		Depth	1.5 mm	Depth	1.5 mm	Depth	1.5 mm	Depth	1.5 mm
		Width	0.5 mm	Width	2 mm	Width	4 mm	Width	8 mm
		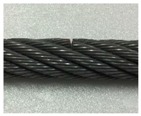		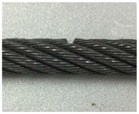		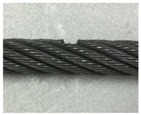		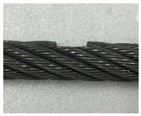	
Wire #4	Upper	Damage #4-2		Damage #4-4		Damage #4-6		Damage #4-8	
		Location	150 mm	Location	350 mm	Location	550 mm	Location	750 mm
		Depth	2 mm	Depth	2 mm	Depth	2 mm	Depth	2 mm
		Width	1 mm	Width	3 mm	Width	6 mm	Width	9 mm
		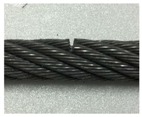		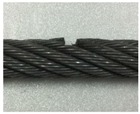		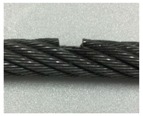		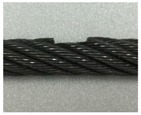	
	Under	Damage #4-1		Damage #4-3		Damage #4-5		Damage #4-7	
		Location	50 mm	Location	250 mm	Location	450 mm	Location	650 mm
		Depth	2 mm	Depth	2 mm	Depth	2 mm	Depth	2 mm
		Width	0.5 mm	Width	2 mm	Width	4 mm	Width	8 mm